# Reactive oxygen species derived from NADPH oxidase as signaling molecules regulate fatty acids and astaxanthin accumulation in *Chromochloris zofingiensis*

**DOI:** 10.3389/fmicb.2024.1387222

**Published:** 2024-04-29

**Authors:** Yi Yuan, Tiantian Zhao, Weizheng Gao, Wenqi Ye, Yuling Chen, Dongzhe Sun, Zhao Zhang

**Affiliations:** ^1^Ministry of Education Key Laboratory of Molecular and Cellular Biology, Hebei Collaborative Innovation Center for Eco-Environment, Hebei Research Center of the Basic Discipline of Cell Biology, College of Life Sciences, Hebei Normal University, Shijiazhuang, China; ^2^School of Life Sciences, Hebei University, Baoding, China

**Keywords:** NADPH oxidase, ROS, fatty acid, astaxanthin, transcriptomic analysis, WGCNA analysis, *Chromochloris zofingiensis*

## Abstract

Abiotic stresses can increase the total fatty acid (TFA) and astaxanthin accumulation in microalgae. However, it remains unknown whether a unified signal transduction mechanism exists under different stresses. This study explored the link between nicotinamide adenine dinucleotide phosphate (NADPH) oxidase-derived reactive oxygen species (ROS) and the accumulation of fatty acids and astaxanthin in *Chromochloris zofingiensis* under three abiotic stresses. Results showed significant increases in fatty acid, astaxanthin, and ROS levels under nitrogen deficiency, phosphorus deficiency, and high-salinity stress. The introduction of the NADPH oxidase inhibitor diphenyleneiodonium (DPI) decreased the content of these components. This underscores the pivotal role of NADPH oxidase-derived ROS in the accumulation of fatty acid and astaxanthin under abiotic stress. Analysis of transcriptomes across three conditions following DPI addition revealed 1,445 shared differentially expressed genes (DEGs). Enrichment analysis revealed that biotin, betalain, thiamine, and glucosinolate may be important in stress responses. The heatmap demonstrated that DPI notably suppressed gene expression in the fatty acid and carotenoid biosynthesis pathways. Our findings underscore the pivotal role of NADPH oxidase-derived ROS in the accumulation of fatty acid and astaxanthin under abiotic stresses.

## 1 Introduction

Microalgae respond rapidly to environmental changes and can accumulate large amounts of bioactive compounds such as proteins, fatty acids, vitamins, and pigments in a short time, which makes them ideal sources for the production of energy, medicine, food, and feed, including biodiesel, DHA, EPA, beta-carotene, and astaxanthin (Maity et al., 2014; Goh et al., 2019; Sun et al., 2019; Chen et al., 2020). Among them, fatty acids and carotenoids are the typical stress-induced valuable substances in microalgae. Numerous studies have attempted to elucidate the metabolic mechanisms underlying fatty acid and carotenoid accumulation under abiotic stresses. High light intensity, nutrient deficiency (nitrogen, phosphorus, and sulfur), and high salinity are the most commonly used strategies for promoting fatty acid and carotenoid accumulation in microalgae (Mao et al., 2018; Liu et al., 2019; Chen et al., 2020; Hang et al., 2020). Under stress conditions, the growth of algal cells generally tends to decrease, and the metabolism of algal cells shifts toward lipid synthesis and accumulation (Nanda et al., 2021). Carotenoid metabolism s also enhanced, resulting in a significant increase in the production of secondary carotenoids. For instance, astaxanthin, is a secondary carotenoid with powerful antioxidant properties that helps cells resist unfavorable environments (Kubo et al., 2022). However, whether a universal signal transduction mechanism exists under different stress conditions remains unclear. Reactive oxygen species (ROS) are by-products of oxygen metabolism and are considered important signaling molecules in algal cells. In particular, ROS can regulate defense responses under adverse conditions (Waszczak et al., 2018; Signorelli et al., 2019). However, excess ROS can cause oxidative damage, leading to apoptosis (Baxter et al., 2014). Studies have speculated that ROS have a potential relationship with fatty acid and carotenoid biosynthesis and are positively correlated with lipid content under nutrient-deprived conditions (Seo et al., 2019; Roy et al., 2021; Liu et al., 2023). In *Chlorella sorokiniana*, nitrogen limitation and hydrogen peroxide (H_2_O_2_) treatment induced ROS elevation, upregulated the expression of lipogenic genes (mitogen-activated protein kinase, heteromeric acetyl-CoA carboxylase beta subunit, β-ketoacyl-ACP synthase III, and diacylglycerol acyltransferase), and promoted lipid synthesis (Wang L. et al., 2023). In *Haematococcus pluvialis*, ROS treatment and abiotic stress treatment can significantly increase astaxanthin production (Cray and Levine, 2022). ROS can be generated by multiple sources, including the mitochondrial electron transport chain, electron transport chain of chloroplast photosystem I, nicotinamide adenine dinucleotide phosphate (NADPH) oxidase, and other organelles associated with ROS production (Shi et al., 2017). However, the source of ROS, which plays a key role in abiotic stress-induced fatty acid and carotenoid accumulation in microalgae, remains unknown.

Plant NADPH oxidase, also known as the respiratory burst oxidase homolog (RBOH), is a homolog of the mammalian macrophage NADPH oxidase gp91phox (Kaur et al., 2014). ROS produced by plant NADPH oxidase can act as secondary messengers in numerous biological processes, such as plant growth and development, plant reproduction, hormone regulation, and biotic and abiotic stress responses. Modulation of NADPH oxidase activity improves seed germination and root growth tolerance to water and oxidative stress (Wang Q. et al., 2023). Application of an NADPH oxidase inhibitor reversed the negative effects of chromium (Cr) stress (Singh et al., 2023). The structure of the plant NADPH oxidase is conserved and comprises two EF-hands structures, six α-transmembrane helical domains, one flavin adenine dinucleotide (FAD) domain, and one NADPH domain. Two EF chiral structures in the N-terminal region of *Arabidopsis thaliana AtRBOH* bind to calcium ion (Ca^2+^) to activate *AtRBOH*, which catalyzes the transfer of electrons from NADPH (electron donor) to O_2_ (electron acceptor) via FAD, membranes, and heme to generate O_2_^–^, which is involved in ROS production (Kaya et al., 2019). Diphenyleneiodonium (DPI) is an NADPH oxidase inhibitor that inhibits ROS production by NADPH oxidase (Foreman et al., 2003). Inhibition of NADPH oxidase by DPI treatment can lead to abnormal pollen tube growth in spruce plants (Liu et al., 2009). Following the treatment of *A. thaliana* tissues with DPI under high-intensity light or heat stress, it was observed that ROS production was hindered by the inhibition of NADPH oxidase, ultimately preventing systematic tissue adaptation (Suzuki et al., 2013). Although NADPH oxidase-derived ROS have been extensively studied in higher plants, the universal signaling role in microalgae under abiotic stresses remains unknown.

To fill this gap in the relationship between NADPH oxidase-derived ROS and fatty acid and carotenoid accumulation under abiotic stress, we investigated the effects of the NADPH oxidase inhibitor DPI on the cellular ROS level, total fatty acid (TFA) content, and carotenoid content in *Chromochloris zofingiensis* under three abiotic stress conditions, including nitrogen deficiency, phosphorus deficiency, and high salinity stress. In addition, comparative transcriptome analysis was conducted to provide a theoretical basis for future studies related to TFA metabolism in microalgae and potential loci for molecular biological modifications of microalgae. Our study aimed to reveal the correlation between intracellular ROS levels and lipid accumulation and carotenoids, and elucidate the role of NADPH oxidase-derived ROS in the response to abiotic stress in *C. zofingiensis*.

## 2 Materials and methods

### 2.1 Strains and culture conditions

*Chromochloris zofingiensis* from the American Typical Culture Conservation Center (ATCC, Rockville, MD, USA) was used in this study. To activate the *C. zofingiensis* cells, 10 ml of seed solution was inoculated into a 500 ml conical flask (with filter membrane) containing 100 ml of Kuhl growth medium (Gao et al., 2023). Glucose was used as the carbon source at a concentration of 5 g L^–1^ and incubated in the dark at 150 rpm in an incubator at 25°C. After incubation for 4 days, when the cells were in the late exponential stage, the active cells were inoculated into 100 ml of Kuhl medium and kept in darkness for 4 days to improve the viability of the cells. The resulting culture was used as a seed for further growth. In order to investigate the influence of NADPH oxidase-derived ROS on fatty acid accumulation, the seed culture was gathered, resuspended in 0.5 g L^–1^ dry weight (DW), and cultivated in the dark under nitrogen deficiency (KNO_3_^–^ deficient medium, denoted as -N group), phosphorus deficiency (PO_4_^–^ deficient medium, denoted as -P group), and high-salinity stress (additional 20 g L^–1^ NaCl, denoted as HS group). To study the effect of the NADPH oxidase inhibitor, 25 μg ml^–1^ DPI (DPI uses DMSO for dissolution) added to nitrogen-deficient medium was denoted as the -N + DPI group, 5 μg ml^–1^ DPI added to phosphorus deficient medium and high salt medium, and denoted as the -N + DPI, -P + DPI, and HS + DPI groups, respectively. Fresh Kuhl medium without any additional chemicals was used as the control. After 6 h of induction, fresh algal cells were collected to measure the intracellular ROS and Ca^2+^ levels. After 4 days of induction, fresh algal cells were collected and lyophilized in a vacuum freeze dryer to measure TFA and carotenoid contents. To rule out the cytotoxic effect of DMSO, 100 μl DMSO was added to group C for a validation experiment (Supplementary Figure 1).

For comparative transcriptome sequencing, samples were collected 6 h after induction. The samples were centrifuged at 7,000 × *g* for 1 min at 4°C and the cells were rapidly frozen in liquid nitrogen. Stored at −80°C for subsequent experiments. All measurements were performed in triplicates.

To make a clear description, the experimental design was drawn and showed in Supplementary Figure 2.

### 2.2 Evaluate microalgal growth status by cell count

*Chromochloris zofingiensis* cells were cultured in different stress-treated media, and cell culture suspensions from 0, 24, 48, 72, 96, 120, and 144 h were taken for cell counting using hemocytometer. The cell suspension at each time point was first blown and mixed with a pipette gun. Then 10 μl of cell suspension was aspirated and quickly punched along the lower edge of the coverslip of the hemocytometer. Waiting for the microalgal cells to fall into the grid of the hemocytometer, they were counted using a light microscope and the total number of cells was calculated. All measurements were performed in triplicate.

### 2.3 Evaluate microalgal growth status by DW

After 4 days of cultivation 5 ml of algal cells were collected from different culture conditions, and the dry cell weight was measured. Each sample was washed three times with distilled water and filtered onto pre-weighted Whatman 0.47 mm filter paper sheets using a diaphragm pump, followed by vacuum drying at 50°C for 4 h to evaporate the water from the cells, and the difference in the filter paper was the DW of the cells. All measurements were performed in triplicate.

### 2.4 Measurement of cellular ROS level

Intracellular ROS levels were measured with a ROS assay kit (Beyotime Institute of Biotechnology, China). This kit uses intracellular ROS to oxidize diethyl 2′,7′-dichlorofluorescein diacetate (DCFH-DA) to the highly fluorescent compound dichlorofluorescein (DCF), and the fluorescence of DCF was measured to calculate intracellular ROS level (Cash et al., 2007). Dilution of DCFH-DA with fresh culture medium solution was carried out in 1:1,000 to 10 μmol L^–1^ final concentration. The cells were collected and incubated at 25°C for 20 min. The mixture was inverted every 5 min during incubation to ensure sufficient contact between the probe and the cells. At the end of the incubation period, the cells were washed three times with the fresh culture medium to remove any residual DCFH-DA which did not enter the cells. Finally, the fluorescence intensity was measured with a Tecan (Switzerland, Spark Cyto) enzyme marker at 480 and 530 nm respectively. Meanwhile, fluorescence imaging of ROS was observed using an inverted microscope (Axio Vert.A1). All measurements were performed in triplicates.

### 2.5 Measurement of cellular calcium ions level

Intracellular Ca^2+^ levels were measured using a Fluo-4 Calcium Assay Kit (Beyotime Institute of Biotechnology, China). The Ca^2+^ fluorescent probe Fluo-4 AM is almost non-fluorescent; once it enters the cell, it is hydrolyzed by intracellular esterases and binds to Ca^2+^, which emits green fluorescence. Cells were inoculated in 96-well black multi-well plates according to the kit instructions, and the number of cells per well was maintained at 100–10,000. The cells were then centrifuged at 1,000 × *g* for 5 min, the supernatant was discarded, the cells were washed with PBS, and incubated for 20 min at 25°C in the dark. Finally, the fluorescence intensity was measured at 490 nm excitation and 525 nm emission wavelengths using a Tecan enzyme marker. All measurements were performed in triplicates.

### 2.6 Carotenoid extraction and measurement

After lyophilization, 20 mg of algal powder was thoroughly crushed with a mortar. Extraction was performed using chromatographic-grade acetone, and the algal residue was collected by centrifugation at 10,000 rpm at 4°C for 5 min after shaking. The algal residue was extracted twice with chromatographic acetone until it became colorless. The filtered acetone extracts were combined and the pigments were dried with nitrogen before being dissolved in 1 ml of chromatographic acetone. The resulting solution was then filtered with an organic filter membrane of 0.22 μm and stored in a brown sample bottle (Zhang Z. et al., 2017). The carotenoid content was quantified using a Waters 2695 HPLC system. Measurements according to the method proposed by Ip and Chen (2005). The chemical standards of β-carotene, lutein and astaxanthin were purchased from Sigma (St. Louis, MO, USA). All measurements were performed in triplicate.

### 2.7 Total fatty acid content analysis

Lyophilized cells (20 mg) were incubated with 2 ml of 1% methanol sulfate solution (v/v, containing 0.01% BHT) and 0.5 ml of heptadecanoic acid at 85°C for 2.5 h for fatty acid methyl ester (FAMEs). The reaction mixture was then shaken for 30 min. After cooling, 1 ml of 0.75% NaCl solution was added and the FAMEs were extracted using 2 ml of chromatographic-grade hexane. The supernatant was collected via centrifugation at 5,000 × *g* for 5 min, and the samples were subsequently nitrogen blown using a pressurized nitrogen concentrator. The sample was filtered through a disposable microporous filter (13 mm × 0.22 μm). FAMEs were performed by 7890A gas chromatography (Agilent, USA). All measurements were performed in triplicates.

### 2.8 Comparative transcriptome analysis

After 6 h of cultivation under each culture condition, 50 ml of algal cell cultures was collected in sterile enzyme-free centrifuge tubes, centrifuged at 5,000 × *g* for 3 min at 4°C, the supernatant was discarded, and the precipitated cells were rapidly frozen in liquid nitrogen. The TRIzol reagent (Invitrogen) was used to extract total cell RNA, and the genomic DNA was removed with DNase I (Code No. 9108/9109, Beijing, China). To determine the quality of the extracted RNA, a 2100 Bioanalyzer (Agilent, Santa Clara, CA, USA) and NanoDrop-2000 (NanoDrop Technologies, Wilmington, DE, USA) were used. RNA-seq transcriptome libraries were prepared using the TruSeq RNA sample preparation kit (Illumina, San Diego, CA, USA) with 1 μg of total RNA. Under the effect of reverse transcriptase, single-stranded cDNA was synthesized by reversing the mRNA as a template using random primers, followed by two-stranded synthesis to form a stable double-stranded structure. A cDNA library with a cDNA target fragment of 300 bp was chosen and amplified via PCR on 2% low-range super agarose, and 15 cycles of PCR were carried out using Phusion DNA polymerase (NEB) to produce the final library. RNA-seq data are publicly available through the Genome Sequence Archive under the accession number CRA012296.

Following the retrieval of gene read counts, samples were scrutinized for differentially expressed genes (DEGs) between groups. Genes that showed differential expression were identified, and their functions were explored. The difference analysis software was: DESeq2, the screening threshold was: | log_2_ (fold change) (log_2_FC)| ≥ 1 and *p*-adjust < 0.05, when a gene met both conditions, then the gene was considered as a DEGs (Wang et al., 2010). To control for calculated false positive rates, four multiplex tests (Bonferroni, Holm, BH, and BY) were used to correct P values, and in general, the Gene Ontology (GO) function and Kyoto Encyclopedia of Genes and Genomes (KEGG) pathway were considered significantly enriched when the corrected *P* ≤ 0.05. All measurements were performed in triplicate.

### 2.9 Weighted correlation network analysis

Weighted correlation network analysis (WGCNA) is a systems biology method used to characterize gene association patterns between different samples by clustering highly correlated genes into modules (Langfelder and Horvath, 2008). After background correction and normalization of gene expression data, abnormal and small variation genes were filtered and outlier samples were excluded. The strength of the correlation between processed genes followed a scale-free distribution, as demonstrated by the coefficient matrix constructed using Spearman’s linear correlation coefficients. The key parameter β value for the WGCNA analysis was then determined. Clustering of similar gene expression patterns into modules. Association between the gene network and the phenotype of interest, as well as the hub genes in the network.

### 2.10 Quantitative real-time PCR analysis

Real-time fluorescence quantitative PCR (RT-qPCR) was used to verify the transcriptome data using a real-time fluorescence quantitative PCR system (Bio-Rad, Hercules, CA, USA). Ten genes were selected and Primer Premier 5 was used to design primers (Supplementary Table 1). One microgram of RNA in each sample was reverse-transcribed into cDNA using the QuantScript RT Kit (Tiangen, China), and then amplified by RT-qPCR. Three PCR were performed for each sample. Evaluation of relative gene expression by 2^–ΔΔCT^ method (Livak and Schmittgen, 2001).

### 2.11 Statistical analysis

All experiments were performed with three biological replicates. Variability is shown as mean and standard deviation (SD). The experimental plots were plotted using Origin 2023. The data were analyzed quantitatively and statistically by one-way analysis of variance (ANOVA) using SPSS version 19.0. The differences were statistically significant (*P* < 0.05).

## 3 Results and discussion

### 3.1 Effect of NADPH oxidase-derived ROS on cells, DW, ROS, and Ca^2+^ of *C. zofingiensis* under abiotic stress conditions

**FIGURE 1:**
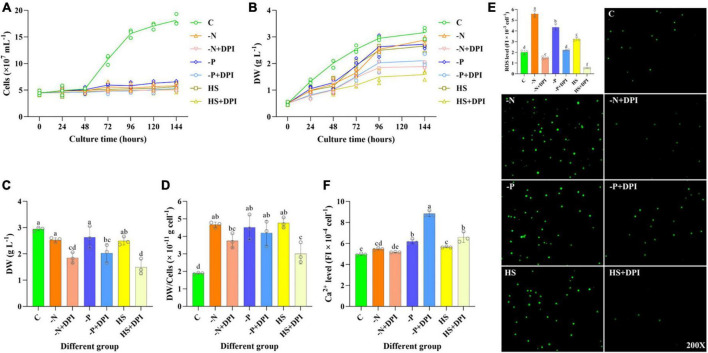
Growth status, ROS and Ca^2+^ levels of *Chromochloris zofingiensis*. **(A)** *C. zofingiensis* growth curve. **(B)** Dry weight (DW) change of *C. zofingiensis* from 0 to 6 days. **(C)** DW of *C. zofingiensis* cultured for 4 days. **(D)** Average DW per cell of *C. zofingiensis* cultured for 4 days. **(E)** ROS level of *C. zofingiensis* in control, stress, and DPI group after 6 h of induction. The 200× means that the inverted microscope is 200 times larger. **(F)** Ca^2+^ level of *C. zofingiensis* in control, stress, and DPI group after 6 h of induction. The error bars show the standard deviation. Different letters (a, b, c, d, e) indicate a significant difference between groups (*P* < 0.05).

The growth curves of *C. zofingiensis* under different abiotic stresses with or without DPI addition were firstly measured. As the cultivation time increased, the cell number in the control group increased at a faster rate, but cell division was inhibited to varying degrees in all treatment groups (Figure 1A). Different with the cell number, the DW of -N, -P, and HS groups were not significantly different from the control group, but the DW of all the DPI-added groups were significantly reduced (Figures 1B, C). The average DW of *C. zofingiensis* cell in DPI addition groups were generally lower than that in stress treatment groups (Figure 1D). Consequently, the DW per cell was significantly decreased by DPI. These suggested that NADPH oxidase derived ROS might be a key regulator in abiotic stressed induced storage components accumulation. After 4 days of cultivation, DW was slightly reduced in the -N, -P, and HS groups compared with that in the control group (Figure 1C). In addition, the addition of DPI significantly decreased DW in the -N, -P, and HS groups by 27.11%, 23.04%, and 40.11%, respectively. This result is in agreement with previous study, which showed that *C. zofingiensis* DW gradually decreased with an increase in DPI concentration compared to the control (Sun and Zhang, 2021).

The intracellular ROS levels were measured at 0, 6, 24, 48, and 96 h of cultivation, which were firstly increased at 6 h of cultivation, and then gradually decreased along the cultivation (Supplementary Figure 3). As the incubation time increased, the ROS level gradually decreased, so we focused on analyzing the ROS level after 6 h of cultivation. Compared with the control group, the ROS level increased significantly under -N, -P, and HS conditions at 6 h, with respective ratios of 2.78, 2.16, and 1.61 times higher than the control (Figure 1E). This was consistent with the previous studies that microalgal ROS are elevated under stress conditions (Cui et al., 2020; Liu et al., 2023). Specifically, the addition of DPI significantly decreased ROS levels by 73.32%, 49.06%, and 83.13% under -N, -P, and HS conditions, respectively (Figure 1E). Meanwhile, the imaging results of ROS levels were observed by using an inverted microscope to aid in verifying the reliability of the above data (Figure 1E). The -N, -P, and HS groups showed stronger fluorescence level compared to the control group and the treatment group with DPI addition, indicating that -N, -P, and HS increased the intracellular ROS level, but the addition of DPI inhibited ROS production. These results suggest that NADPH oxidase is a universal producer of intracellular ROS in *C. zofingiensis* under stressful conditions.

Apart from ROS, Ca^2+^ is also involved in a variety of signaling pathways in microalgae, affecting a variety of metabolic pathways (Sagi and Fluhr, 2001; Chen et al., 2014). Ca^2+^ may be involved in the regulation of lipid accumulation, as it was previously shown that lipid accumulation increased after the addition of 100 mM CaCl_2_ in *Chlamydomonas reinhardtii* and a synergistic effect was observed between NaCl and CaCl_2_ (Hang et al., 2020). In higher plants, ROS production leads to the activation of plasma membrane Ca^2+^ channels, which in turn increases the cytosolic free Ca^2+^ concentration and regulates root growth (Pei et al., 2000). Thus, we measured the cytosolic free Ca^2+^ concentration to determine whether it is another universal signaling molecule in *C. zofingiensis* under abiotic stress. As shown in Figure 1F, intracellular Ca^2+^ levels were slightly higher under -N, -P, and HS stress conditions compared to the control group, whereas TFA content showed the same trend, indicating that intracellular Ca^2+^ levels under stress conditions may be correlated with fatty acid accumulation. However, the addition of DPI significantly increased the intracellular Ca^2+^ levels under phosphorus deficiency and high-salinity conditions. From the above results, it can be inferred that NADPH oxidase mediates ROS generation and triggers cytoplasmic calcium signaling; however, Ca^2+^ is not regulated in the same way as NADPH oxidase-derived ROS under different stresses.

### 3.2 Effect of NADPH oxidase-derived ROS on fatty acid accumulation under abiotic stresses

**FIGURE 2:**
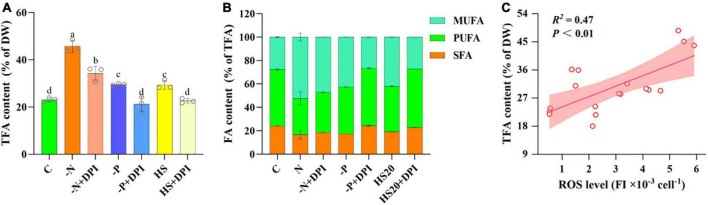
Fatty acids content. **(A)** TFA of *Chromochloris zofingiensis* in control, stress, and DPI group after 4 days of cultivation. **(B)** Percentage of fatty acids to TFAs content of *C. zofingiensis* after 4 days of cultivation. **(C)** Relationship between the ROS level and TFA content of *C. zofingiensis* under different culture conditions. Lines are linear fit with Pearson correlation coefficient (*R*^2^). Shaded areas indicate 95% confidence bands. The error bars show the standard deviation. Different letters (a, b, c, d) indicate a significant difference between groups (*P* < 0.05).

Increased fatty acid accumulation is a typical response of microalgae to abiotic stresses (Yu et al., 2018; Shetty et al., 2019; Latsos et al., 2020; Nanda et al., 2021). Our experiments showed that the intracellular TFA content of *C. zofingiensis* was approximately 23.07% of DW in the control group, whereas it accumulated to 45.75%, 29.59%, and 29.36% of DW under -N, -P, and HS conditions, which was 1. 98-, 1. 28-, and 1.27-fold of that in the control group, respectively (Figure 2A). This suggests that fatty acid accumulation in *C. zofingiensis* can be enhanced by multiple stressors, and the addition of the NADPH oxidase inhibitor DPI significantly reduced TFA content from 45.75% to 34.26%, 29.36% to 21.23%, and 29.59% to 22.60% under -N, -P, and HS conditions, respectively. Given the decrease in ROS levels in the DPI-treated groups, it is reasonable to speculate that NADPH oxidase-derived ROS may play an important role in regulating fatty acid metabolism in *C. zofingiensis* under various abiotic stresses.

Compared to the control group, the percentage of saturated fatty acids (SFA) decreased from 24.26% to 16.79%, 17.45%, and 19.28%, under -N, -P, and HS stress conditions, respectively, and the percentage of unsaturated fatty acids (USFA) increased from 75.74% to 83.21%, 82.55%, and 80.72%, respectively. The percentage of monounsaturated fatty acids (MUFA) increased from 27.59% to 52.50%, 42.59%, and 41.96%, whereas the percentage of polyunsaturated fatty acids (PUFA) decreased from 48.15 to 30.71%, 39.97%, and 38.76%, respectively (Figure 2B and Table 1). It has been shown that nitrogen starvation could increase the SFA content but decrease the USFA in microalgae (*Dunaliella tertiolecta*, *Dunaliella salina*, *Chlorella minutissima*, and *Desmodesmus* sp. MCC34) (Nagappan and Kumar, 2021). On the contrary, the USFA and MUFA percentages of *C. zofingiensis* were significantly increased under high salt stress (Vitali et al., 2023). These results indicate that the relative abundance of fatty acids in microalgae is highly dependent on the culture conditions (Liu et al., 2016; Mao et al., 2018; Zhang et al., 2021). It is worth noting that in our study, the addition of DPI reversed the variation trends of SFA, MUFA, and PUFA percentages under three stresses.

**TABLE 1:** The percentage of fatty acid profiles (% of TFA) of *Chromochloris zofingiensis* in different stress treatment groups.

Fatty acid component	Control	-N	-N + DPI	-P	-P + DPI	HS	HS + DPI
C16:0	22.48 ± 0.09	16.37 ± 3.36	18.68 ± 0.14	17.45 ± 0.02	23.04 ± 0.35	19.28 ± 0.08	21.42 ± 0.05
C16:1	2.03 ± 0.29	3.66 ± 1.99	2.52 ± 0.12	1.50 ± 0.05	1.94 ± 0.30	1.66 ± 0.24	2.12 ± 0.05
C16:2	6.80 ± 0.19	5.12 ± 0.85	5.22 ± 0.07	4.85 ± 0.04	6.59 ± 0.61	5.68 ± 0.09	7.37 ± 0.07
C16:3	1.73 ± 0.06	0.49 ± 0.26	0.72 ± 0.01	1.83 ± 0.05	2.65 ± 0.26	1.37 ± 0.04	1.79 ± 0.01
C18:1	25.57 ± 1.08	48.84 ± 3.60	44.54 ± 0.10	41.09 ± 0.06	24.47 ± 0.17	40.30 ± 0.26	24.98 ± 0.03
C18:2	26.36 ± 0.71	17.27 ± 6.87	20.60 ± 0.08	21.35 ± 0.06	23.97 ± 0.10	22.04 ± 0.11	26.88 ± 0.10
C18:3	12.03 ± 0.12	7.22 ± 0.66	7.09 ± 0.05	8.31 ± 0.09	14.66 ± 0.21	9.14 ± 0.08	12.50 ± 0.16
SFA	24.26 ± 0.25	16.79 ± 3.64	18.68 ± 0.14	17.45 ± 0.02	24.30 ± 0.44	19.28 ± 0.08	22.89 ± 0.06
USFA	75.74 ± 0.25	83.21 ± 3.64	81.32 ± 0.14	82.55 ± 0.02	75.70 ± 0.44	80.72 ± 0.08	77.11 ± 0.06
MUFA	27.59 ± 0.42	52.50 ± 3.33	47.06 ± 0.08	42.59 ± 0.11	26.41 ± 0.15	41.96 ± 0.19	27.09 ± 0.06
PUFA	48.15 ± 0.67	30.71 ± 5.50	34.26 ± 0.14	39.97 ± 0.08	49.29 ± 0.36	38.76 ± 0.27	50.02 ± 0.01

Palmitic acid (C16:0), oleic acid (C18:1), linoleic acid (C18:2), and α-linolenic acid (C18:3) were the main fatty acids in *C. zofingiensis*, accounting for more than 85% of the TFAs. Under the three stress treatment conditions, the ratio of C18:1 increased significantly compared to the control, where it was 48.84%, 39.13%, and 40.30% of TFA under -N, -P, and HS, respectively, which was significantly higher than the 25.57% TFA of the control group. This is in agreement with Zhang et al. (2021) that the production of C18:1 increased under nitrogen deficiency stress conditions. C18:1 can balance oxidative stability and low temperature performance, and its high abundance is beneficial to improve the quality of biodiesel. DPI does restore the stressed cultures to control for the most part, but it is quite interesting to note that C18:1 in the nitrogen starved DPI culture remains elevated, which contributes to a reduction in the PUFA content. In contrast to C18:1, the content of C16:2 and C18:3 in TFAs was significantly lower under stress conditions than in the control group. Similarly, the addition of DPI reversed this increase and significantly decreased C18:1 content. However, the C16:2 and C18:3 contents showed a slight increase after adding DPI. Compared to the control group, the trends of fatty acid ratio changes under the three stresses tended to be consistent, and the similar trends of all three stresses were reversed after the addition of DPI. Thus, inhibition of NADPH oxidase could reverse the fatty acid changes that arise from abiotic stress in *C. zofingiensis*. This suggests that NADPH oxidase-derived ROS may play a key role in the regulation of TFA content and fatty acid ratio under abiotic stress.

The relationship between ROS and fatty acid biosynthesis was verified by Pearson correlation analysis (Table 2). The Pearson correlation coefficients of intracellular TFA content and ROS levels were 0.909, 0.910, and 0.960 under -N, -P, and HS conditions, respectively. Thus, it can be concluded that the accumulation of TFAs was strongly correlated with intracellular ROS levels. The accumulation of TFAs in *C. zofingiensis* increased with an increase in intracellular ROS levels within a certain range (Figure 2C). Several studies have indicated that ROS levels may be correlated with lipid accumulation. For instance, the accumulation of stress induced lipid in *Tetradesmus obliquus* KMC24 has been linked to elevated intracellular levels of ROS (Roy et al., 2021). In *C. reinhardtii*, high salt concentration could inhibit the growth of microalgae and induce three main stresses: osmotic, ionic (salt), and oxidative stress (Khona et al., 2016). Excessive cadmium has also been shown to elevate intracellular the concentration of lipid and ROS levels in *Monoraphidium* sp. QLY-1 simultaneously (Zhao et al., 2019). However, our results further confirmed that NADPH oxidase-derived ROS levels under different stress conditions were positively correlated with TFA content. These results indicate that NADPH oxidase-derived ROS may act as universal signaling molecules to regulate fatty acid accumulation under abiotic stress conditions.

**TABLE 2:** Correlation coefficients of ROS and TFA in the stress and DPI groups.

Index	All	-N/-N + DPI	-P/-P + DPI	HS/HS + DPI
ROS	0.687**	0.909*	0.910*	0.960**

The symbol “*” denotes significant at the 0.05 level, while “**” indicates significant at the 0.01 level.

### 3.3 Effect of NADPH oxidase-derived ROS on carotenoids content under abiotic stresses

**FIGURE 3:**
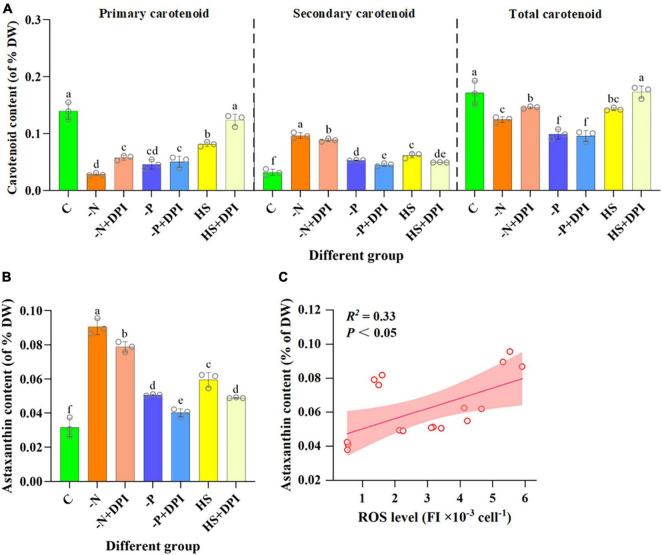
Carotenoid content after 4 days of cultivation. Carotenoid **(A)**, astaxanthin **(B)** content of *Chromochloris zofingiensis* after 4 days of induction under different conditions. **(C)** Relationship between the ROS level and astaxanthin content of *C. zofingiensis* under different culture conditions. Lines are linear fit with Pearson correlation coefficient (*R*^2^). Shaded areas indicate 95% confidence bands. The error bars show the standard deviation. Different letters (a, b, c, d, e, f) indicate a significant difference between groups (*P* < 0.05).

Apart from TFAs, secondary carotenoids are another typical component that would accumulate under stress conditions. Astaxanthin is a valuable secondary carotenoid produced by *C. zofingiensis* (Zhang K. et al., 2017). ROS is been considered an important regulator of astaxanthin accumulation in microalgae cells (Cray and Levine, 2022). In the present study, the carotenoid content of *C. zofingiensis* was measured 96 h after induction. All primary carotenoids, including lutein and β-carotene, showed a severe decrease in response to -N, -P, and HS stress conditions (Figure 3A). Previous studies have shown that under nitrogen deficiency and high light stress, intracellular levels of lutein and β-carotene decrease (Zhang et al., 2019). Additionally, secondary carotenoids, such as astaxanthin and adonixanthin, were present in trace amounts under normal conditions, but significantly accumulated under stressed conditions (Figure 3B). The -N, -P, and HS stress also increased astaxanthin accumulation by 223.64%, 73.07%, and 110.39%, respectively, compared with the control group. However, secondary carotenoids content decreased significantly after the addition of DPI under stress conditions (Figure 3B). Since DPI may block the production of NADPH oxidase-derived ROS under stress conditions, fewer signaling molecules were produced; thus, the astaxanthin content was significantly reduced by the addition of DPI under stress conditions. The correlation between NADPH oxidase-derived ROS levels and astaxanthin biosynthesis in *C. zofingiensis* under stress conditions was also verified using Pearson correlation analysis (Table 3). The Pearson correlation coefficients between intracellular astaxanthin accumulation and ROS levels in *C. zofingiensis* under -N, -P, and HS conditions were 0.866, 0.911, and 0.961, respectively. The Pearson’s correlation coefficients for the different groups were greater than 0.7. These results indicated that the accumulation of intracellular astaxanthin in *C. zofingiensis* was closely related to its intracellular NADPH oxidase-derived ROS levels under stress conditions. Astaxanthin content increased with an increase in intracellular ROS levels within a certain range (Figure 3C). These results further confirmed that ROS levels, especially NADPH oxidase-derived ROS, may have a regulatory effect on astaxanthin accumulation under abiotic stress conditions.

**TABLE 3:** Correlation coefficients of ROS and astaxanthin in the stress and DPI groups.

Index	All	-N/-N + DPI	-P/-P + DPI	HS/HS + DPI
ROS	0.578*	0.866*	0.911*	0.961**

The symbol “*” denotes significant at the 0.05 level, while “**” indicates significant at the 0.01 level.

### 3.4 Differential expression genes and enrichment analysis under abiotic stresses

In order to elucidate the molecular mechanism of NADPH oxidase-derived ROS in microalgal cell substance synthesis under stress conditions, comparative transcriptome analysis was performed. On the basis of principal component analysis (PCA), the transcriptional profiles of the eight groups in this study were highly reproducible in three replicates (Supplementary Figure 4). Based on the results of expression quantification, differential gene analysis between groups was performed to identify the genes that were differentially expressed of both groups. Log_2_FC was used to express the expression changes in the differential ratio analysis groups, and the number of genes that were down- and up-regulated in each differential ratio analysis group was determined (Supplementary Table 2).

**FIGURE 4:**
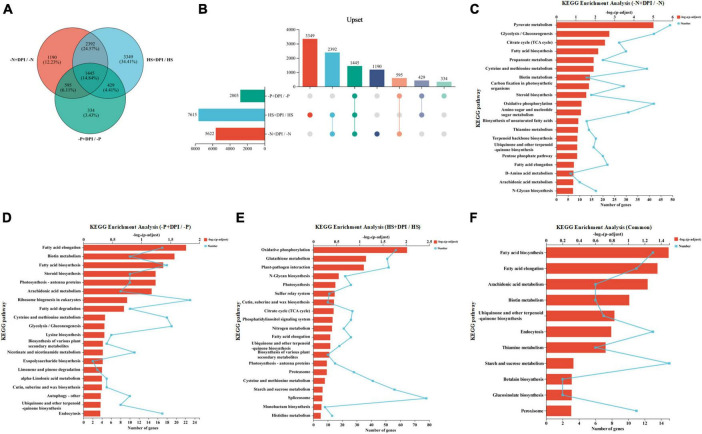
Venn analysis and functional enrichment analysis. **(A)** The number of shared and unique differentially expressed genes at 6 h. Different colored circles represent DEGs sets and the values represent the number of common and unique genes among DEGs sets. **(B)** Upset figure. The horizontal bar chart on the left represents the statistical values of the elements of each set, the individual points in the middle matrix represent the elements specific to a set, the lines between the points represent the intersections specific to different sets, and the vertical bar charts represent the values of the elements of the corresponding intersections, respectively. **(C–F)** KEGG enrichment analysis of the three groups including “-N + DPI/-N,” “-P + DPI/-P,” “HS + DPI/HS,” and “Common.” The vertical coordinate indicates the KEGG pathway, and the lower horizontal coordinate indicates the number of genes in this pathway on the ratio, corresponding to the different points on the fold; the upper horizontal coordinate indicates the significance level of enrichment, corresponding to the height of the bar, where the smaller the false discovery rate and the larger the –log_10_(*p*-adjust) value, the more significantly enriched the KEGG pathway is (*p*-adjust < 0.5).

The DEGs were analyzed across the three abiotic stresses after the addition of DPI, and Venn diagrams were generated. As depicted in Figures 4A, B, there are 5,622, 2,803, and 7,615 DEGs of the “-N + DPI/-N,” “-P + DPI/-P,” and “HS + DPI/HS” groups, respectively. A total of 1,445 DEGs were shared among the three groups. KEGG enrichment analysis was performed on the DEGs of each group and their common DEGs (Figure 4C–F). The “-N + DPI/-N,” “-P + DPI/-P,” and “HS + DPI/HS” gene sets were all enriched for pathways associated with fatty acid biosynthesis and terpenoid quinone biosynthesis (precursors of carotenoid synthesis) (Figures 4C–E). These results indicated that ROS derived from NADPH oxidase would be a regulator of fatty acids and astaxanthin biosynthesis under abiotic stresses. Besides, 11 pathways were enriched in the common DEGs, the first two enriched pathways were the fatty acid biosynthesis and fatty acid elongation pathways, further supported the role of NADPH oxidase-derived ROS in regulating fatty acid biosynthesis under stress conditions (Figure 4F). In addition, the ubiquinone and other terpenoid-quinone biosynthesis pathways were also enriched. Ubiquinone is an important isoprenoid quinone that acts as an antioxidant in plant responses to stress that regulate gene expression and cell signaling (Abby et al., 2020). Terpenoids are a large group of organic molecules found widely in nature, and are a major type of pigment (Lin et al., 2023). Biotin, betalain, thiamine, and glucosinolate biosynthesis pathways, which play crucial roles in plant growth, development, and stress coping, were found to be enriched (Barba-Espin et al., 2018; Wang et al., 2020; Deng et al., 2022; Zhang et al., 2023). Biotin is essential for fatty acid carbon chain extension as it catalyzes the formation of CoA malonate from acetyl-CoA, which is a two-carbon unit supplier. Biotin deficiency alters the intracellular fatty acid composition and decreases SFA synthesis, explaining the reversal of saturated fatty acid reduction after DPI addition under stress conditions. Betalain reduces damage to cell membranes, enzymes, and protein structure and function caused by osmotic water loss under adverse conditions, thus increasing plant resistance to various stress factors. Thiamine plays an integral role as a cofactor in key metabolic reactions in all organisms, including glycolysis, pentose phosphate pathway, and tricarboxylic acid cycle (Goyer, 2010). Previous studies have shown that thiamine and its intermediates, acting as signaling molecules, are involved in systemic acquired resistance in various plant species (Tunc-Ozdemir et al., 2009). Thiamine accumulation was induced by the three stress treatments, thereby reducing oxidative stress damage to microalgal cells. Li et al. (2023) showed that CaCl_2_-HCl electrolyzed water induces calcium signaling to activate NADPH oxidase-derived ROS to regulate the growth and metabolism of glucosinolate. Our analysis of DEGs under stress treatments revealed that inhibition of NADPH oxidase significantly affected glucosinolate biosynthesis. Except for the component biosynthesis, peroxisome related pathway was found to be enriched. The peroxisome is an organelle present in all eukaryotic cells, and its main function is to catalyze fatty acid β-oxidation to break down very long-chain fatty acids (VLCFAs) into short-chain fatty acids. Thus, inhibition of NADPH oxidase-derived ROS under different stress conditions can significantly regulate a range of cellular component synthesis and environmental stress-related pathways, suggesting that NADPH oxidase-derived ROS play an important regulatory role in a variety of stress processes.

We selected ten important genes for RT-qPCR confirm the validity of the comparative transcriptome data. These genes were involved in fatty acid biosynthesis, carotenoid biosynthesis, and the peroxisome pathway. As shown in Supplementary Figure 5, the expression profiles of all selected genes showed changes similar to those observed in the comparative transcriptome analysis. Therefore, transcriptome data were considered plausible.

### 3.5 Typical pathways analysis

To elucidate the role of NADPH oxidase-derived ROS in the regulation of fatty acid and carotenoid accumulation, we analyzed the enriched pathways. Biochemical data showed that fatty acid and carotenoid contents were significantly decreased in all stress treatment groups after the addition of DPI. Moreover, the primary function of peroxisomes is to catalyze fatty acid β-oxidation, which can also affect the fatty acid content. Therefore, we focused our analysis on the biosynthesis of fatty acids, carotenoids, and peroxisome pathways. Detailed information on the genes involved in the biological pathways selected above is provided in Supplementary File 1.

**FIGURE 5:**
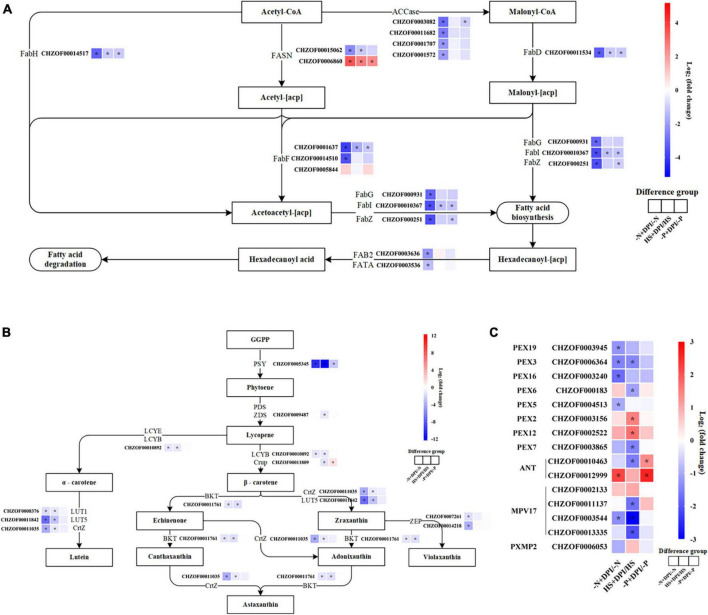
Up-down-regulation of typical pathway genes. The expression changes in the fatty acid biosynthesis pathway **(A)**, carotenoid biosynthesis pathway **(B)**, and peroxisome pathway **(C)**. [Heat map indicates the variation of log_2_FC in the differential comparison analysis groups. Genes are displayed in red (upregulated) and blue (downregulated). The isozymes are ranked in descending order of expression. Significantly differentially expressed genes |log_2_FC| ≥ 1, *P* < 0.05].

Acetyl-CoA carboxylase (*ACCase*) is considered the first key rate-limiting enzyme in the fatty acid biosynthetic pathway. Comparative transcriptome analysis showed that *ACCase* was downregulated by DPI-treatment, especially under nitrogen starvation conditions. Other genes involved in the fatty acid biosynthesis pathway included S-malonyltransferase (*FabD*), 3-oxoacyl-(acyl-carrier-protein) synthase II (*FabF*), 3-oxoacyl-(acyl-carrier-protein) synthase III (*FabH*) and, 3-hydroxyacyl-[acyl-carrier-protein] dehydratase (*FabZ*), and enoyl-(acyl-carrier protein) reductase (*FabI*) (Figure 5A). The biochemical data indicated that large amounts of fatty acids accumulated under various stress conditions, but this accumulation was significantly hindered by the inclusion of DPI. These findings corroborate the molecular viewpoint that NADPH oxidase-derived ROS play a regulatory role in fatty acid synthesis.

In the carotenoid synthesis pathway, β-carotene is the precursor for astaxanthin biosynthesis, followed by hydroxylation and ketolation (Zhou et al., 2019). In bacteria and algae, the synthesis of zeaxanthin from β-carotene is mainly catalyzed by β-carotene hydroxylase (*CrtZ*) and β-carotene ketolase (*BKT*) (Liu et al., 2014). Zeaxanthin is further ketonized to astaxanthin via *BKT*. The addition of DPI under -N, -P, and HS stress conditions down regulated the expression of *CrtZ* and *BKT* (Figure 5B). These transcriptomic results are in agreement with biochemical data, implying that hindering NADPH oxidase activity under stress conditions obstructs ROS signaling, which leads to a decrease in astaxanthin biosynthesis by downregulating the expression of genes related to astaxanthin synthesis.

Peroxisomes are single membrane-covered vesicles present in most eukaryotic cells. Its main function is to participate in lipid metabolism, such as catabolism of long-chain fatty acids. Glutathione S-transferase kappa 1 (*GSTK1*) and superoxide dismutase (*SOD*) have antioxidant effects and are involved in the regulation of plant secondary metabolism, detoxification, and defense (Ferreira et al., 2023). Peroxidase is one of the key enzymes in the enzymatic defense system of plants under adverse conditions, and acts synergistically with *SOD* and catalase (*CAT*) to scavenge excess free radicals in the body, thereby increasing plant resistance (Dazy et al., 2009). The peroxisome gene encoded by peroxidase plays a crucial role in the cellular metabolism and pathogenicity of microalgae (Kato et al., 2022). Our results showed that most peroxidase genes were down-regulated upon inhibition of NADPH oxidase-derived ROS under various stress conditions, such as peroxin-3 (*PEX3*), peroxin-5 (*PEX5*), peroxin-7 (*PEX7*), peroxin-16 (*PEX16*), and peroxin-19 (*PEX19*) genes (Figure 5C). Inhibition of NADPH oxidase-derived ROS led to the suppression of signal transduction and downregulation of peroxidase genes, which in turn weakened the plant defense system. The upregulation of the adenine nucleotide transporter (*ANT*) gene may be due to its role as a reverse transporter that transports adenosine diphosphate (*ADP*) from the cytoplasm to the mitochondrial matrix and adenosine 5′-triphosphate (*ATP*) synthesized by the mitochondrial matrix to the cytoplasm, thus providing energy to the organism. Supplementary Table 3 shows the peroxisomal proteins, in which long-chain acyl-CoA synthesis (*ACSL*), acyl-CoA oxidase (*ACOX*) and 2,4-dienoyl-CoA reductase (*DECR2*) are involved in fatty acid β-oxidation. All three genes were significantly upregulated. This explains the significant decrease in TFA content after the inhibition of NADPH oxidase-derived ROS.

### 3.6 WGCNA analysis

**FIGURE 6:**
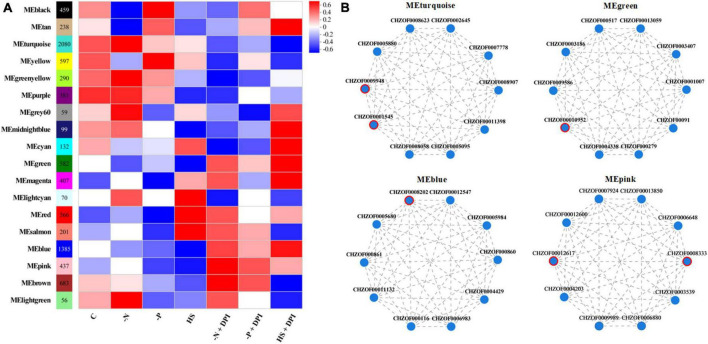
Weighted correlation network analysis (WGCNA). **(A)** Correlation between module and trait. **(B)** Visualization and analysis images of the top 10 hub genes. The genes marked in red circle are key genes.

A variety of abiotic stresses lead to the accumulation of TFAs and carotenoids in *C. zofingiensis*, and transcriptome analysis can be used to determine variations in gene expression levels for fatty acid and carotenoid biosynthesis. However, it is unclear whether a universal regulatory mechanism exists under various stress conditions. To address this, we performed WGCNA and established gene clusters that were highly correlated with phenotypic characteristics. After filtering abnormal and small variant genes, we ensured that the intensity of the correlation effects among processed genes conformed to a scale-free distribution following background correction and normalization. Intracellular genes and genes with similar expression profiles were clustered into modules. The correlation between phenotypes and modules was analyzed to identify the key modules that were significantly regulated by NADPH oxidase under stress conditions. As shown in Figure 6A, 8,749 genes were clustered into 18 modules. Correlation analysis with the phenotypic data revealed that 582 and 1,385 genes in the MEgreen and MEblue modules, respectively, were negatively correlated with stress conditions but positively correlated with the addition of DPI under stress conditions. The MEturquoise module contains 2,080 genes in the MEturquoise module that were positively associated with phenotype under stress conditions, but negatively associated with the addition of DPI under stress conditions. We also searched for modules containing genes involved in fatty acid biosynthesis, carotenoid biosynthesis, and peroxisome-related processes, and found 58 genes in the three pathways. Of these, 39 genes were included in the WGCNA analysis, and more than half of the genes were found to be distributed in the three most abundant clusters (MEturquoise, MEpink, and MEblue). Therefore, we considered the MEturquoise, MEgreen, MEblue, and MEpink modules as key modules. The top 10 hub genes for connectivity within the target modules were screened using BLAST, and the top ten hub genes in the different modules are shown in Figure 6B, and the hub genes relevant to this study are listed in Table 4.

**TABLE 4:** The hub genes of different modules.

Module	Gene ID	Annotated	Degree
MEturquoise	CHZOF0009948	Protein phosphatase PTC7	840.800
	CHZOF0001545	Pyruvate dehydrogenase E2 component	837.534
MEgreen	CHZOF00010952	5′-AMP-activated protein kinase	324.218
MEblue	CHZOF0008202	Mechanosensitive ion channel protein	483.858
MEpink	CHZOF0008333	Methyltransferase	189.934
	CHZOF00012617	Ubiquitin-like 1-activating enzyme E1 A	183.900

Among the key modules screened, protein phosphatase PTC7 (*PPTC7*, CHZOF0009948), 5′-AMP-activated protein kinase (*AMPK*, CHZOF00010952), mechanosensitive ion channel protein (*MSL*, CHZOF0008202), and ubiquitin-like 1-activating enzyme E1 A (*UBLE1A*, CHZOF00012617) are all involved in signal transduction and are important for cells to perform normal physiological functions. Previous research showed that up-regulation of the pyruvate dehydrogenase E2 component (*aceF*) would promote the production of acetyl-CoA in *H. pluvialis*, resulting in an increase of fatty acid accumulation (Zhao et al., 2020). Therefore, the down-regulated expression of *aceF* (CHZOF0001545) by the addition of DPI might be the reason for the decrease fatty acid content under stress conditions. Stimulation of ROS production leads to the activation of *AMPK* (Ohishi et al., 2021). *AMPK* regulates fatty acid biosynthesis through phosphorylation and inactivation by acetyl-CoA carboxylase. CHZOF0008333 is a methyltransferase that converts demethylmenaquinone (*DMKH2*) to menaquinone (*MKH2*) and functions in the plant and microbial electron transport chains. Moreover, cellular signaling pathways primarily involved in nitrogen fixation have been identified in *Klebsiella pneumoniae* (Thummer et al., 2007). *UBLE1A* is the ultimate step in the downstream signaling pathway, which interacts with downstream ubiquitination mechanisms to regulate gene expression. In summary, we propose a plausible signal transduction pathway in which microalgal cells perceive stress and generate ROS signals, which continue to signal downward through phosphorylation and ubiquitination, ultimately resulting in fatty acid and carotenoid biosynthesis. However, the above speculations are only based on histological data, and more work is needed to verify the regulation of NADPH oxidase under stress conditions. In summary, through WGCNA analysis, this study identified six potential core regulatory-related genes in *C. zofingiensis*, providing a framework for future research.

## 4 Conclusion

This study examined the correlation between the generation of ROS by NADPH oxidase and the accumulation of fatty acids and astaxanthin under -N, -P, and HS stress conditions. The results showed that these stresses induced fatty acid and astaxanthin accumulation, whereas treating the plants with DPI under stress conditions reduced ROS generation and significantly decreased fatty acid and astaxanthin accumulation. The correlation between ROS and TFA and astaxanthin content was strong, with Pearson correlation coefficients of over 0.9 and 0.8, respectively. Comparative transcriptome analysis showed that the expression levels of key enzymes involved in the fatty acid and carotenoid biosynthesis pathways were downregulated in the DPI group. Moreover, biotin, betaine, thiamine, and glucosinolate metabolism pathways were enriched in the DPI group under different stress conditions, indicating that these pathways may be regulated by NADPH oxidase-derived ROS and may play important roles in regulating the biosynthesis of TFAs and carotenoids. However, the exact regulatory mechanisms underlying these pathways require further functional verification. In summary, this study proved that NADPH oxidase-derived ROS might play a universal regulatory role in the production of fatty acids and astaxanthin under stress conditions, which suggests that NADPH oxidase as well as its downstream regulating pathways could be promising manipulation points for the production of lipids and carotenoids in microalgae.

## Data availability statement

The datasets presented in this study can be found in online repositories. The names of the repository/repositories and accession number(s) can be found in the article/Supplementary material.

## Author contributions

YY: Data curation, Formal analysis, Investigation, Validation, Writing – original draft, Writing – review & editing. TZ: Data curation, Formal analysis, Investigation, Writing – original draft. WG: Data curation, Formal analysis, Writing – review & editing. WY: Methodology, Writing – review & editing. YC: Data curation, Writing-review & editing. DS: Conceptualization, Supervision, Writing – review & editing. ZZ: Conceptualization, Investigation, Supervision, Writing – review & editing.
