# Supplementary material for: Reactive oxygen species derived from NADPH oxidase as signaling molecules regulate fatty acids and astaxanthin accumulation in Chromochloris zofingiensis
Source: Front Microbiol. 2024 Apr 29;15:1387222. doi: 10.3389/fmicb.2024.1387222 (PMC11089112; doi:10.3389/fmicb.2024.1387222)
Supplement: Supplementary file 2 [file Table_2.DOCX]

***Supplementary Material***

**Supplementary Figures and Tables**


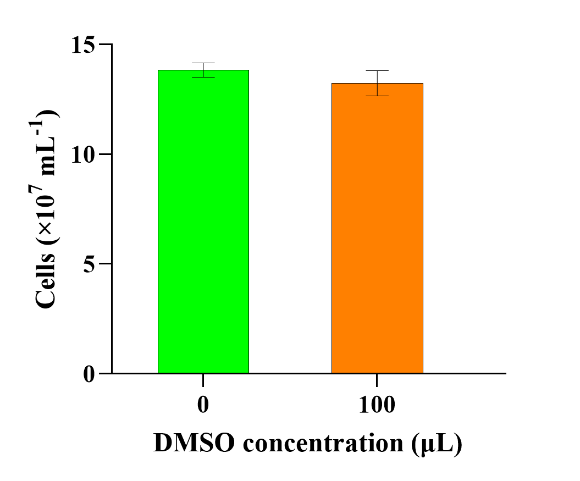


**Figure S1.** **DSMO verification experiment.**


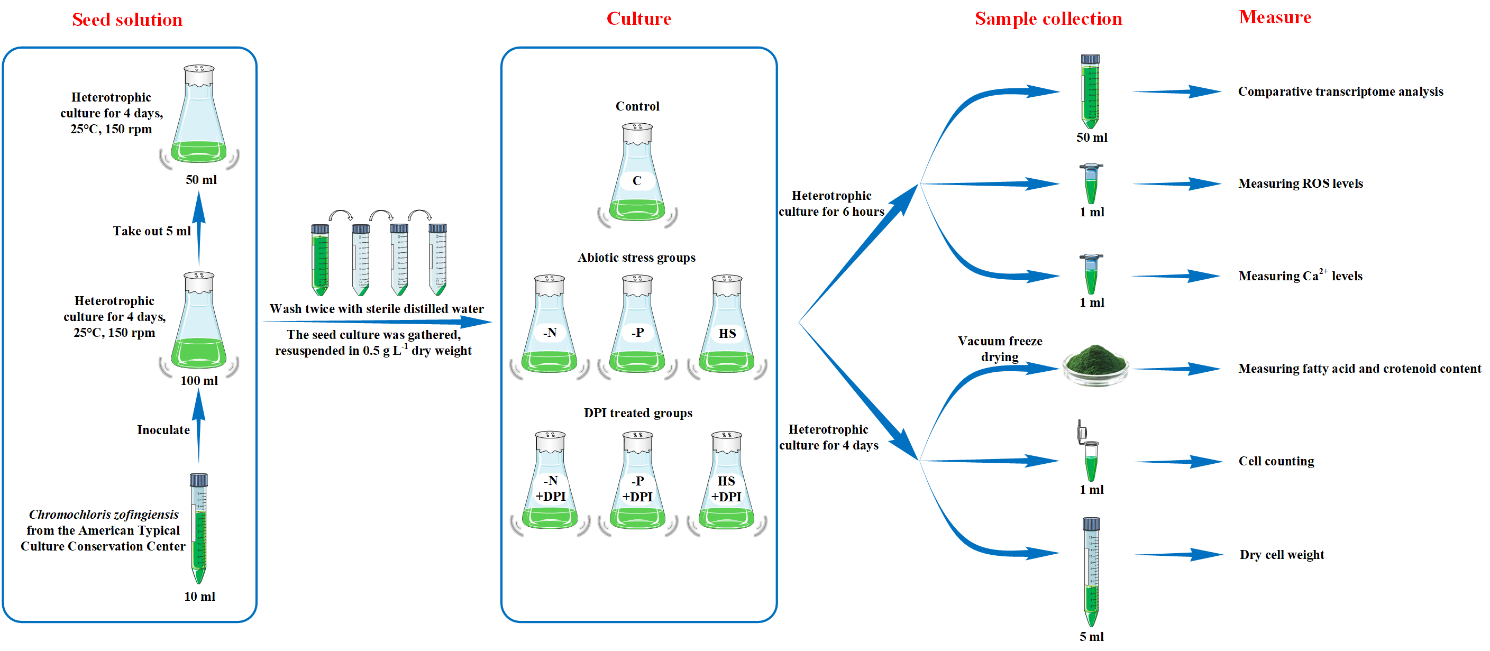


**Figure S2.** ***Chromochloris zofingiensis* culture protocol.**


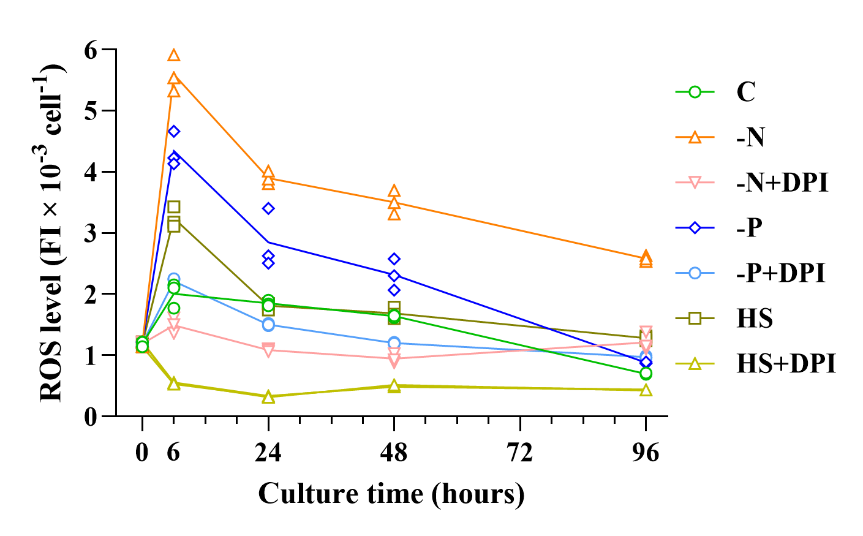


**Figure S3.** **The fluctuation of ROS level in microalgae.**


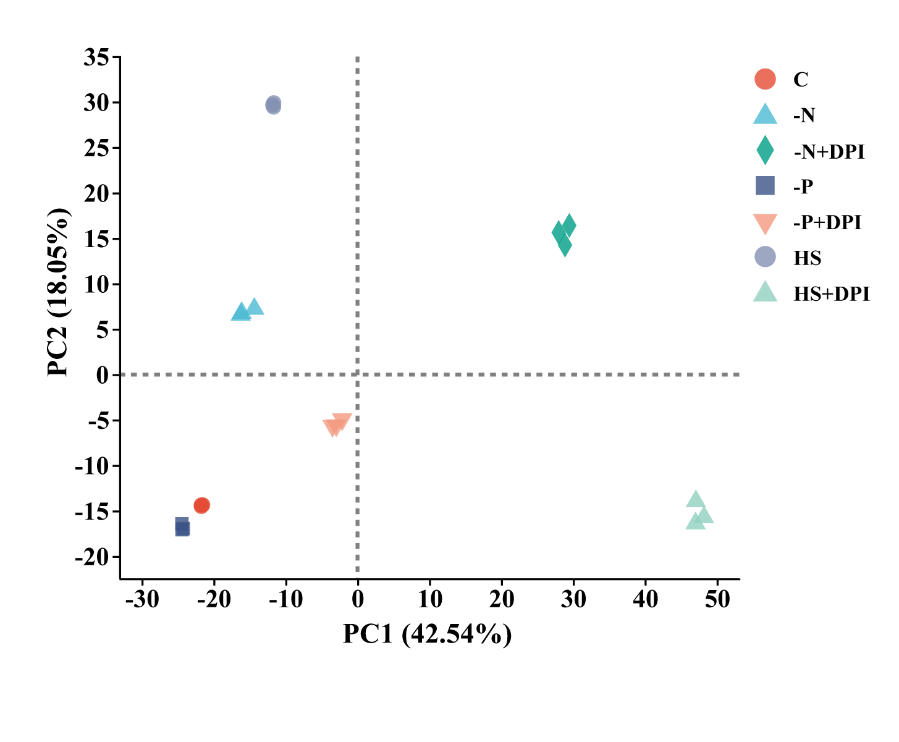


**Figure S4.** **Principal component analysis (PCA).**


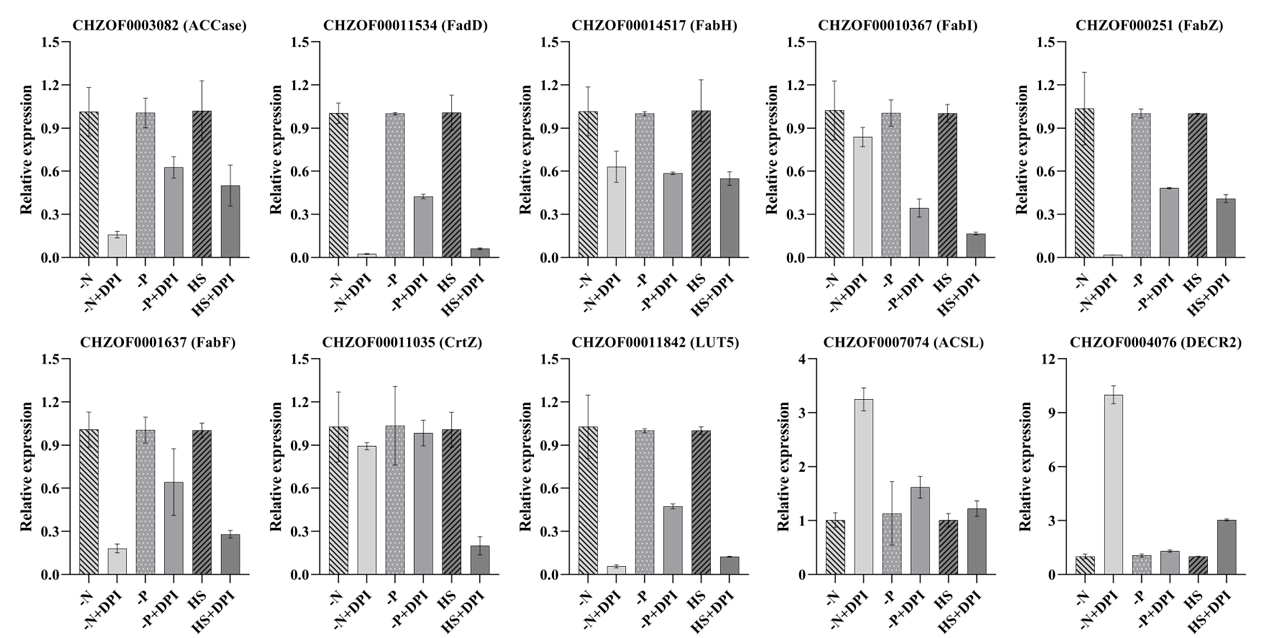


**Figure S5. Expressed genes detected by RT-qPCR after 6 h of incubation.** All measurements were three biological replicates. The error bars show the standard deviation. The 10 key genes are acetyl-CoA carboxylase carboxyl transferase subunit alpha (*ACCase*, CHZOF0003082), [acyl-carrier-protein] S-malonyltransferase (*FabD*, CHZOF00011534), 3-oxoacyl-[acyl-carrier-protein] synthase III (*FabH*, CHZOF00014517), enoyl-[acyl-carrier protein] reductase I (*FabI*, CHZOF00010367), 3-hydroxyacyl-[acyl-carrier-protein] dehydratase (*FabZ*, CHZOF000251), 3-oxoacyl-[acyl-carrier-protein] synthase Ⅱ (*FabF*, CHZOF0001637), beta-carotene 3-hydroxylase (*CrtZ*, CHZOF00011035), beta-ring hydroxylase (*LUT5*, CHZOF00011842), long-chain acyl-CoA synthetase (*ACSL*, CHZOF0007074), and 2,4-dienoyl-CoA reductase [(3E)-enoyl-CoA-producing] (*DECR2*, CHZOF0004076).

**Table S1. Primer sequence of RT-qPCR.**

| **Gene name** | **Forward primer (5’-3’)** | **Reverse primer (5’-3’)** |
| --- | --- | --- |
| *ACCase* | AGTACCGTCTTTGAGCACCC | CCATTCTCCACCAATATCTT |
| *FabD* | GTGGTAGTGTGGCAGGGTGTGACGCT | GTCTTGACAGTGGTCTCCCATTGAAC |
| *FabI* | CAAGCGTTATGCTGGCAATGAA | GGGGTCCCGCAGAGATGGTGTT |
| *FabF* | ATGCCTCCGACTTCCCTACC | ATCACATCAGCTTCCCCTTG |
| *FabH* | TCCCTGCTGCGTCCCTTATT | CCCTGTTCGTGTTCTTATCC |
| *FabZ* | TCAAAGCAGAGGCTGTAGAGGCAA | ACCACACGGTCCACCAGAAGAAAT |
| *CrtZ* | CCACACCTACAACTTCTCAA | GCCAAACACTCCTCCAATCA |
| *LUT5* | ATGAGACGCTGGATGAGTTGATT | CACGTAGCTGCTTGCTGTAGATG |
| *ACSL* | AGGAGATGGATGAGGATGGGT | TGCTTGGTGAGATTAGGACGC |
| *DECR2* | GCGACGACAGGATGTTCTGGATAC | GCAGTTGACGACAATGCTGAGGCT |

**Table S2. DEGs of different groups.**

| **Different group** | **total DEG** | **up** | **down** |
| --- | --- | --- | --- |
| -N+DPI / -N | 5622 | 2775 | 2847 |
| -P+DPI / -P | 2803 | 1656 | 1147 |
| HS+DPI / HS | 7615 | 4087 | 3528 |

**Table S3.** Fold change of peroxisomal protein DEGs common to the analyzed group at 6 hours -N + DPI/ -N, -P + DPI/ -P, and HS + DPI/ HS for three differential comparisons. (The isozymes are ranked in descending order of expression. Significantly differentially expressed genes |Log_2_FC| ≥ 1, *P* < 0.05)

| **Gene name** | **Gene ID** |  | **Log_2_FC** |  |
| --- | --- | --- | --- | --- |
|  |  | **-N + DPI / N** | **-P + DPI / -P** | **HS + DPI / HS** |
| **acyl-CoA oxidase** | CHZOF0007469 | 1.0342 | 1.1566 | 1.0637 |
| **2,4-dienoyl-CoA reductase [(3E)-enoyl-CoA-producing]** | CHZOF0004076 | 2.6536 | 1.7425 | 1.8239 |
| **long-chain acyl-CoA synthesis** | CHZOF0007074 | 2.1066 | 1.2310 | 2.2525 |
|  | CHZOF0005279 | 1.8333 | 1.5672 | 1.5949 |
|  | CHZOF000973 | -4.9364 | -11.0248 | -1.0779 |
| **fatty acyl-CoA reductase** | CHZOF0001582 | -3.1502 | -1.9826 | -1.2555 |
|  | CHZOF0002305 | -7.6719 | 1.4654 | -3.0524 |
| **superoxide dismutase** | CHZOF0002713 | 1.0299 | -2.0023 | 1.0256 |
| **glutathione S-transferase kappa 1** | CHZOF0009936 | 3.1324 | 2.2729 | 3.1478 |
| **dehydrogenase/reductase SDR family member 4** | CHZOF000129 | 3.6911 | 1.5341 | 2.2765 |
